# Bibliometric analysis of antimalarial drug resistance

**DOI:** 10.3389/fcimb.2024.1270060

**Published:** 2024-02-12

**Authors:** Jialu Zhang, Muhammad Shahbaz, Muhammad Ijaz, Huimin Zhang

**Affiliations:** ^1^ Shandong University of Traditional Chinese Medicine, College of Pharmacy, Jinan, China; ^2^ Shandong Academy of Chinese Medicine, Institute of Chinese medicine analysis, Jinan, China; ^3^ Department of Radiology, Qilu Hospital Affiliated to Shandong University, Jinan, China; ^4^ Research Center for Sectional and Imaging Anatomy, Digital Human Institute, School of Basic Medical Science, Shandong University, Jinan, Shandong, China; ^5^ The Faculty of Medicine, Qilu Institute of Technology, Jinan, China; ^6^ Department of Pharmacology, School of Pharmaceutical Science, Shandong University, Jinan, China

**Keywords:** bibliometric analysis, antimalarial drug, resistance, anti-malarial, CiteSpace

## Abstract

**Background:**

Malaria has always been a serious infectious disease prevalent in the world. Antimalarial drugs such as chloroquine and artemisinin have been the main compounds used to treat malaria. However, the massive use of this type of drugs accelerates the evolution and spread of malaria parasites, leading to the development of resistance. A large number of related data have been published by researchers in recent years. CiteSpace software has gained popularity among us researchers in recent years, because of its ability to help us obtain the core information we want in a mass of articles. In order to analyze the hotspots and develop trends in this field through visual analysis, this study used CiteSpace software to summarize the available data in the literature to provide insights.

**Method:**

Relevant literature was collected from the Web of Science Core Collection (WOSCC) from 1 January 2015 to 29 March 2023. CiteSpace software and Microsoft Excel were used to analyze and present the data, respectively.

**Results:**

A total of 2,561 literatures were retrieved and 2,559 literatures were included in the analysis after the removal of duplicates. An irrefutable witness of the ever-growing interest in the topic of antimalarial drug resistance could be expressed by the exponentially increased number of publications and related citations from 2015 to 2022, and its sustained growth trend by 2023. During the past 7 years, USA, Oxford University, and David A Fidock are the country, institution, and author with the most publications in this field of research, respectively. We focused on the references and keywords from literature and found that the research and development of new drugs is the newest hotspot in this field. A growing number of scientists are devoted to finding new antimalarial drugs.

**Conclusion:**

This study is the first visual metrological analysis of antimalarial drug resistance, using bibliometric methods. As a baseline information, it is important to analyze research output published globally on antimalarial drug resistance. In order to better understand the current research situation and future research plan agenda, such baseline data are needed accordingly.

## 1 Introduction

Malaria is a vector-borne infectious disease caused by *Plasmodium falciparum*, *Plasmodium vivax*, *Plasmodium malariae*, *Plasmodium ovale*, and *Plasmodium knowlesi* and transmitted by *Anopheles mosquitoes*. There are two proven effective strategies to combat malaria (White, 2004). One is to block the *Anopheles mosquitoes* from approaching humans, by blocking the transmission route of *Anopheles mosquitoes* and using insecticide-equipped mosquito nets. The other is to use antimalarial drugs for case management. In 2006, the World Health Organization (WHO) recommended the use of artemisinin-based combination therapies (ACTs) as first-line treatment for falciparum malaria (Eastman and Fidock, 2009). If ACTs fail in Southeast Asia and other regions, there will be no fully effective drug capable of completely replacing them in the future. As the new antimalarial drugs are unlikely to become generally available within the next few years, active measures should be given the highest priority to preserve the current antimalarials.

Widespread drug use leads to antimalarial drug resistance, which is an inevitable risk factor in malaria treatment. This risk cannot be disregarded, particularly with unregulated drug use. Early detection and proper medical intervention can effectively lower malaria mortality rates and prevent the emergence of *Plasmodium* resistance (Landier et al., 2018; Schultz et al., 2023). Over the last few years, antimalarial drug resistance has posed a significant challenge to worldwide malaria control initiatives, especially in the Greater Mekong subregion (Ménard et al., 2018). There are alarming early reports of disturbing mutations in other regions too (Anantabotla et al., 2019; Hussien et al., 2020; Narh et al., 2020; Uwimana et al., 2020; Balikagala et al., 2021; Uwimana et al., 2021; Conrad et al., 2023; Fola et al., 2023; Mihreteab et al., 2023). The global prevalence of *Plasmodium* infection and deaths declined prior to the outbreak, and healthcare services were compromised due to the public health emergencies that exacerbated the common health crisis (Bhatt et al., 2015; McLean et al., 2018; Kiarie et al., 2022). In accordance with the prognosis of the WHO, the insufficient prevention, diagnosis, and treatment of the illness is expected to lead to the doubling of malaria-related fatalities during the pandemic (Norman et al., 2022). According to the 2022 World Malaria Report, there were 247 million cases of malaria globally in 2021, an increase from 245 million cases in 2020. The number of deaths attributed to the disease decreased from 625,000 in 2020 to 619,000 in 2021 (Fact sheet about malaria, n.d). Among available antimalarials, factors that contribute include parasite mutation rates, total parasite load, drug strength, adherence to treatment guidelines, dosing appropriateness, pharmacokinetic properties, inadequate drug contact due to falsified drugs, and poor-quality antimalarials that may lead to or eliminate resistance (Shibeshi et al., 2020). In 2021, amid antimalarial drug resistance, the African region has contributed to 95% of the total global malaria cases and 96% of all deaths related to malaria, with children under five accounting for 80% of all malaria-related deaths in Africa. While artemisinin-based malaria treatment continues to be highly effective in Africa with no evidence of complete resistance, the mounting genetic mutations linked to artemisinin resistance may ultimately result in enhanced overall artemisinin resistance as well as selective pressure on partner drugs (Owoloye et al., 2021). Therefore, continuous monitoring of treatment efficacy and genetic surveillance across Africa is imperative.

Regular monitoring of drug efficacy is necessary to enhance treatment strategies in malaria-endemic countries and ensure early detection and response to drug resistance. The WHO recommends surveillance of antimalarial drug resistance every 2 years to control the spread of antimalarial drug resistance. The predominant techniques employed to oversee antimalarial drug resistance encompass drug efficacy studies, *in vitro*/*in vivo* studies of malaria parasites, and molecular studies that evaluate recognized markers of antimalarial drug resistance (Ariey et al., 2014; Ménard et al., 2016; Chen et al., 2021). To identify antimalarial drug resistance, specific gene mutations and molecular markers associated with antimalarial drug resistance are utilized. Detection of antimalarial drug resistance serves as a useful monitoring mechanism to enhance the control of *Plasmodium* susceptibility. Bibliometrics is a qualitative and quantitative visualization study of the retrieved literature, involving the application of mathematical and statistical methods to scholarly publications. Researchers evaluate research trends and research priorities within the field by analyzing literature databases, which have extensive applications in the pharmaceutical field. This study aimed to visually analyze the research status and development trends of the antimalarial drug resistance, by means of bibliometrics and knowledge mapping.

## 2 Materials and methods

The study employed Web of Science Core Collection (WOSCC) as the literature search database. The retrieved literature was analyzed for research trends, references, and keywords with the aid of CiteSpace and Microsoft Excel. Relevant literature from 1 January 2015 to 29 March 2023 was included, with the date of publication as the selection criterion. The search terms used were “(TS= (Antimalarial Drug) AND TS=(Resistance))”.

## 3 Introduction to software used for bibliometric analysis

CiteSpace (6.2.R1) was utilized for bibliometric analysis, a tool created by Professor Chen Chaomei. The most current version of CiteSpace can be downloaded from the official website and no longer requires the JAVA environment. Its primary function is to visually and analytically represent information found in the academic literature within a given field. The primary objective of visualizing knowledge domains is to identify and track their development by illustrating scientific collective networks within scientific domains, such as scientific networks, social networks of co-authors, citation networks, and networks of collaborators. CiteSpace achieves this by representing relations between scholars or publications through network nodes and connectivity, with the size of a node proportional to the publication output. The publication date is indicated by the color of each node. Technical terms are explained upon their first use. Mediator centrality is assigned to every node in a network, determining the probability of each shortest path passing through it. Mediated centrality is defined for each node in the network to determine the probability that any shortest path in the network passes via the node. Mediator nodes with high centrality may be found at the center of two significant clusters or sub-networks, hence the term mediator. CiteSpace illustrates nodes with high mediator centrality as purple rings, with the thickness representing the magnitude of the value of mediator centrality. The co-cited literature and keywords were clustered using the log-likelihood ratio (LLR) method. The silhouette (*S*) value denotes the mean contour value of the cluster. It is generally accepted that clustering is justifiable when *S* > 0.5 and persuasive when *S* > 0.7.

For this research, CiteSpace was used with the following specific parameters: time slice spanned January 2015 to March 2023; year slice interval was 1 year; term source used included title, abstract, author, and keywords; node type encompassed author, institution, country, keyword, literature, cited author, and cited journal; and the top 50 articles were compiled based on the selection criteria.

Microsoft Excel was utilized to construct tables that illustrate annual national publication trends.

## 4 Results

A total of 2,561 papers related to antimalarial drug resistance in the WOSCC were searched, 2,559 papers were shortlisted after excluding duplicates using CiteSpace, and all papers were included in the analysis after excluding duplicates. From 1 January 2015 to 29 March 2023, a total of 2,561 articles with the subject terms “antimalarial drug” and “resistance” were retrieved from the WOSCC, consisting of the following article types: Article (1,979), Article; Book Chapter (6), Article; Early Access (33), Article; Proceedings Paper (7), Correction (2), Editorial Material (31), Meeting Abstract (31), News Item (1), Review (462), Review; Book Chapter (5), and Review; Early Access (4).

**Figure 1:**
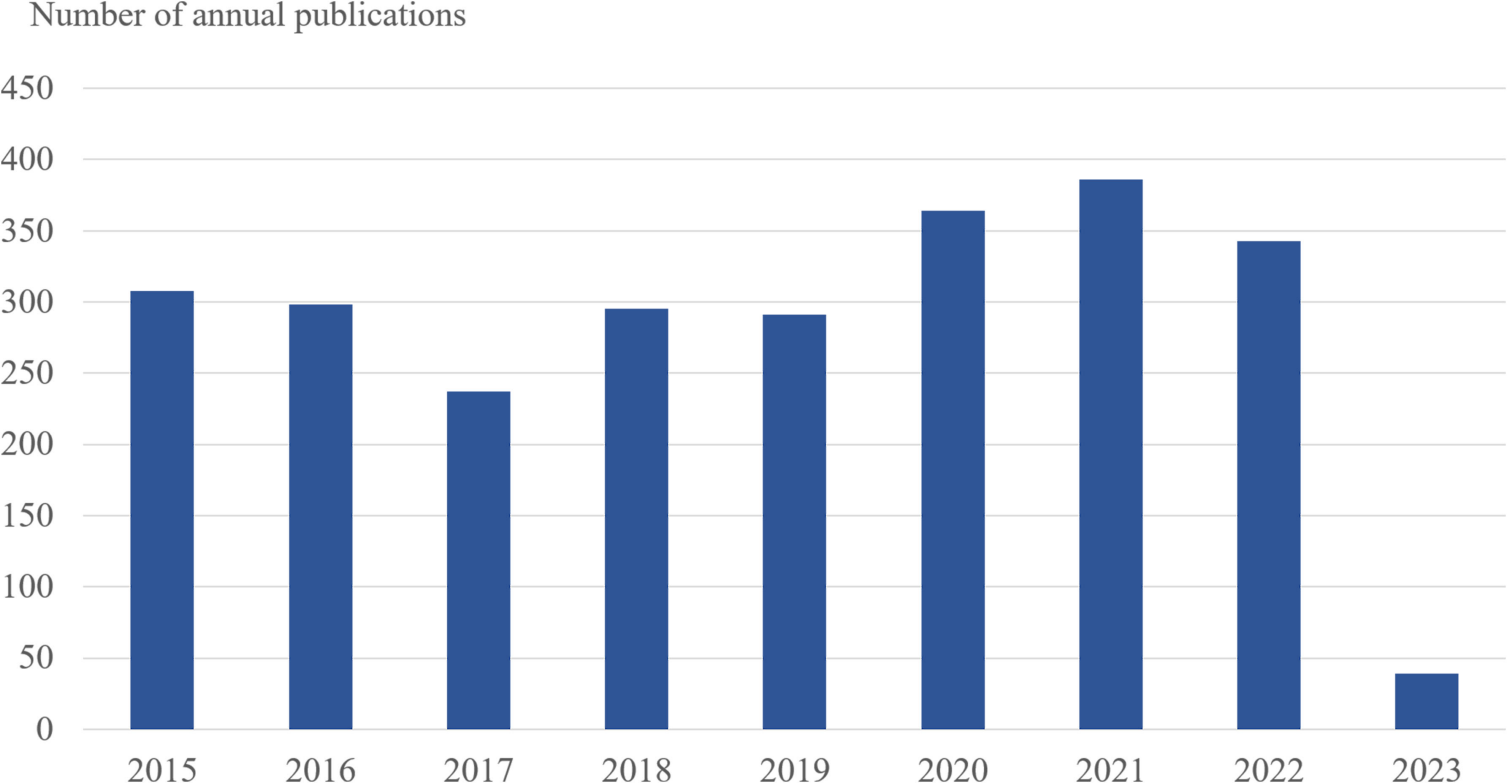
The number of annual publications on antimalarial drug resistance research between 2015 and 2023. The horizontal coordinates represent the year of publication, and the vertical coordinates represent the number of publications.

There were 386 papers in 2021, compared to 363 in 2020, 343 in 2022, and approximately 300 in other years, with the largest amount of literature published (**Figure 1**). In general, the rise in the number of academic papers on malaria may be linked to growing apprehension about the emergence of resistance genes in Africa over the past few years.

### 4.1 Analysis of the cooperation network of sending countries

**Figure 2:**
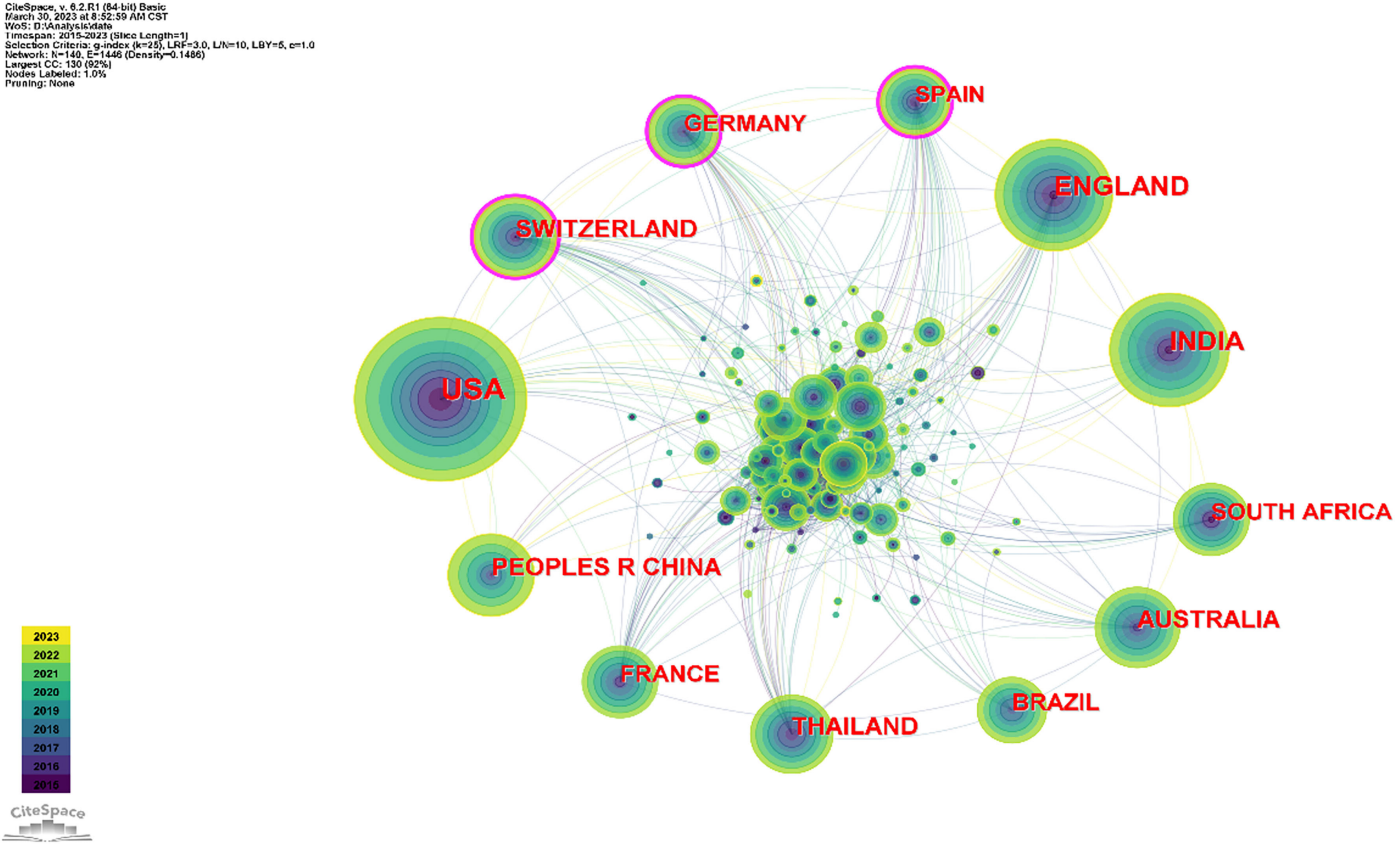
Map illustrating networks of cooperation between countries. Labels with purple circles denote centrality values >0.1, revealing Switzerland, Germany, and Spain as the three countries with the highest centrality. The size of each circle corresponds to the number of countries with which a country cooperates. The largest circle in the diagram represents the USA, indicating its high level of cooperation with other countries.

In order to understand the research hotspots in the analysis of antimalarial drug resistance in various countries, visual analysis was carried out in each country. As **Table 1** and **Figure 2** indicate, the top 10 countries with published papers were USA (744), India (403), England (382), Australia (232), China (217), Thailand (199), Switzerland (197), France (176), South Africa (170), and Germany (154). For centrality, the top ranking is Germany (0.14) followed by Switzerland (0.12), Spain (0.12), Sudan (0.08), and Bangladesh (0.07) (**Table 1**).

**Table 1:** Top 10 publication counts and country-wise centralities.

Ranking	Count	Country	Ranking	Centrality	Country
1	744	USA	1	0.14	Germany
2	403	India	2	0.12	Spain
3	382	England	3	0.12	Switzerland
4	232	Australia	4	0.08	Sudan
5	217	P.R. China	5	0.07	Bangladesh
6	199	Thailand	6	0.07	Denmark
7	197	Switzerland	7	0.07	Malaysia
8	176	France	8	0.07	Vietnam
9	170	South Africa	9	0.06	Colombia
10	154	Germany	10	0.05	Austria

### 4.2 Network analysis of publishing partner institutions

**Figure 3:**
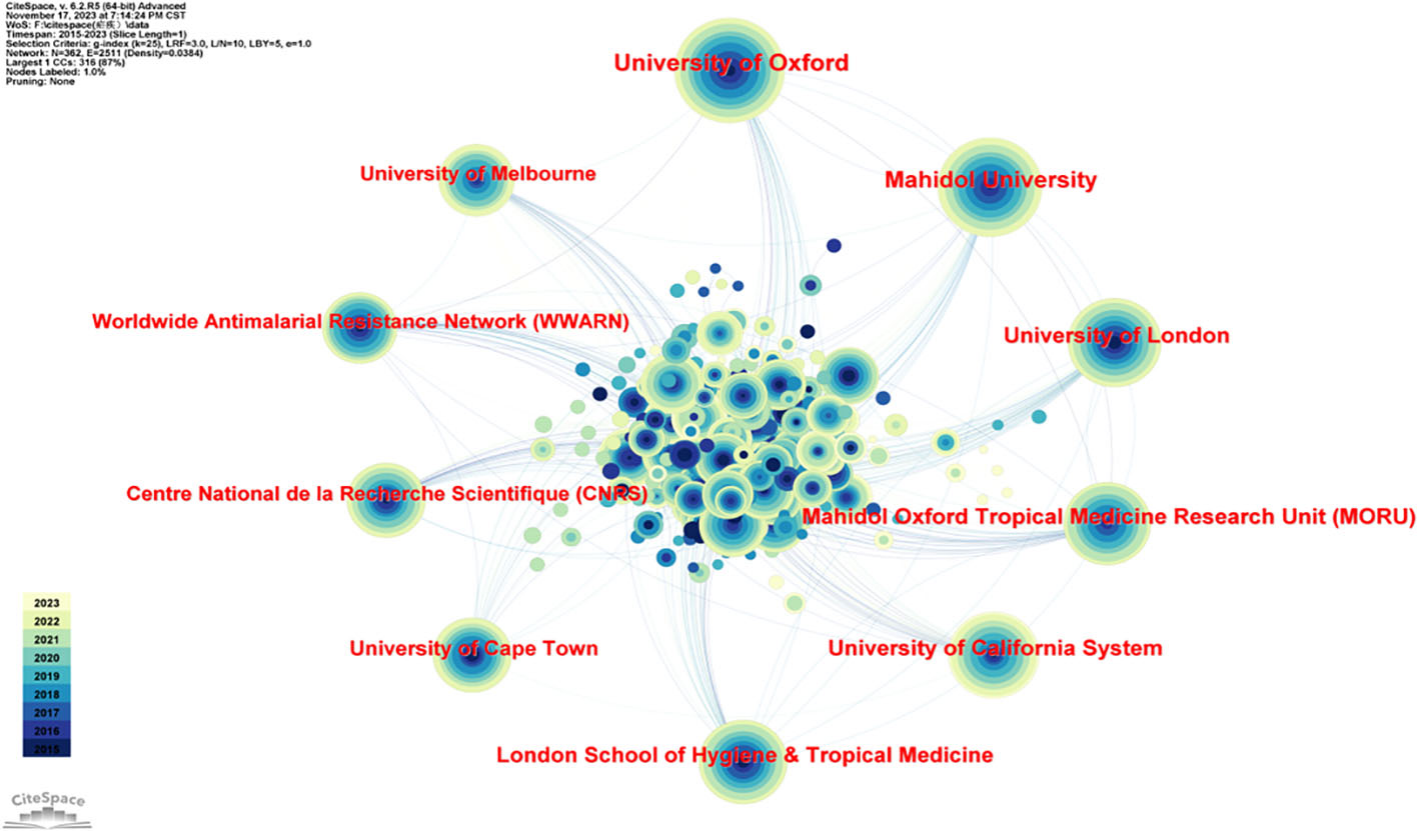
Diagram of the network of cooperation between institutions. The five most prominent institutions are the University of Oxford (England, 186), Mahidol University (Thailand, 168), the University of London (England, 127), the University of California (USA, 122), and Mahidol Oxford Tropical Medicine Research Unit (MORU) (Thailand, 122).

Many institutions and universities from different countries around the world have contributed to this research field. As shown in **Table 2** and **Figure 3**, the top 5 institutions were the University of Oxford (England, 186), Mahidol University (Thailand, 168), the University of London (England, 127), the University of California (USA, 122), and Mahidol Oxford Tropical Medicine Research Unit (MORU) (Thailand, 122). The top 5 institutions with the highest centrality were Columbia University (USA, 0.09), Harvard University (USA, 0.08), Swiss Tropical & Public Health Institute (Switzerland, 0.07), Mahidol University (Thailand, 0.05), and the University of Basel (Switzerland, 0.05).

**Table 2:** Top 10 publication counts and institution-wise centralities.

Ranking	Count	Institutions	Ranking	Centrality	Institutions
1	186	University of Oxford	1	0.09	Columbia University
2	168	Mahidol University	2	0.08	Harvard University
3	127	University of London	3	0.07	Swiss Tropical & Public Health Institute
4	122	Mahidol Oxford Tropical Medicine Research Unit (MORU)	4	0.05	Mahidol University
5	122	University of California System	5	0.05	University of Basel
6	118	London School of Hygiene & Tropical Medicine	6	0.05	Le Reseau International des Instituts Pasteur (RIIP)
7	91	University of Cape Town	7	0.05	Universite Paris Cite
8	90	Centre National de la Recherche Scientifique (CNRS)	8	0.04	University of Oxford
9	89	Worldwide Antimalarial Resistance Network (WWARN)	9	0.04	University of Cape Town
10	87	University of Melbourne	10	0.03	University of California System

### 4.3 Analysis of author network

**Figure 4:**
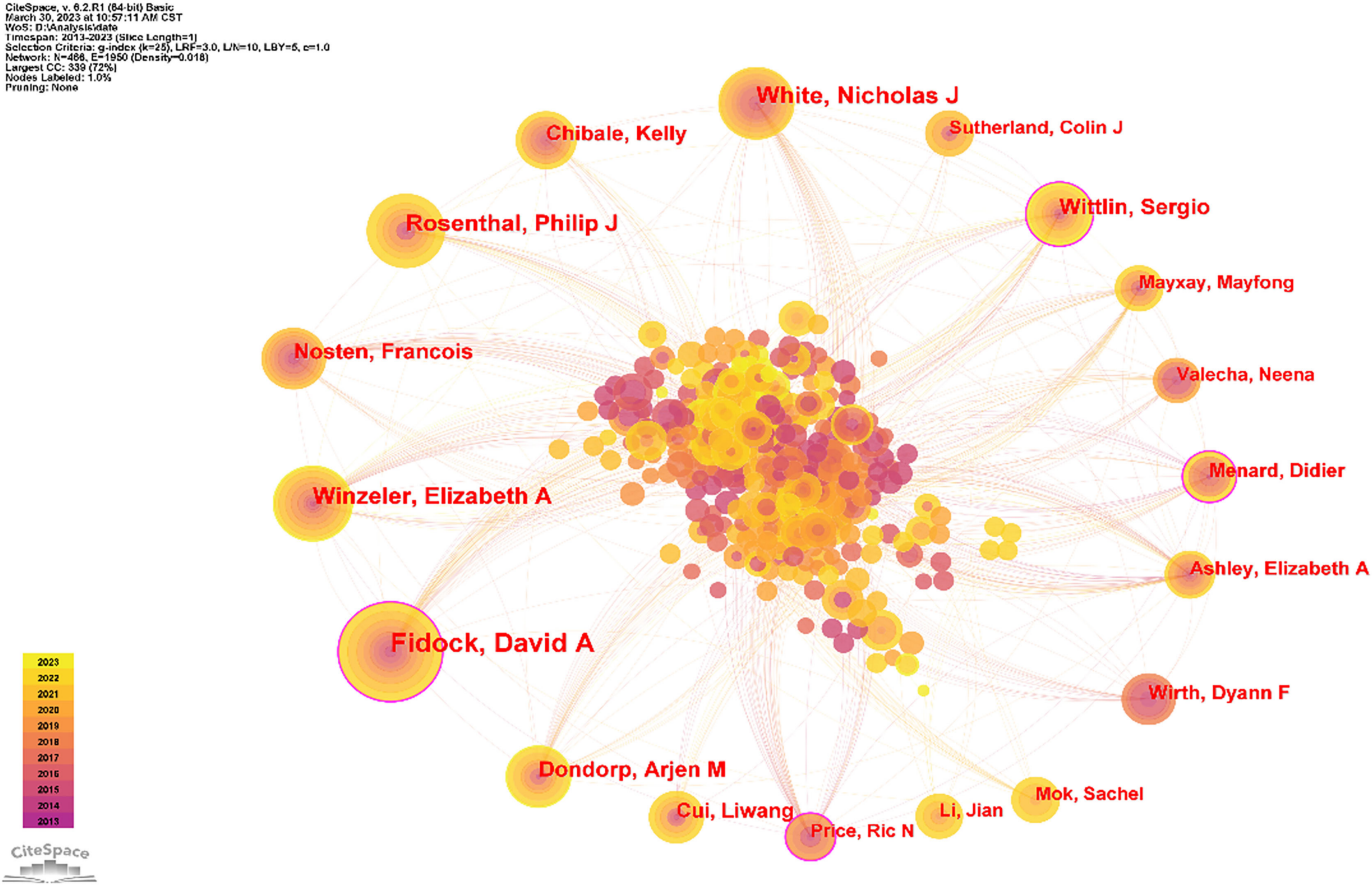
Author collaboration network diagram. As shown in the figure, David A. Fidock is the most collaborative and the most central author. Secondly, Wittlin Serglo’s article may be helpful for research in this direction.

The author partnership network was analyzed to provide information for finding industry giants and seeking partners. As **Table 3** and **Figure 4** show, the top 10 authors were David A. Fidock (74), Nicholas J. White (44), Elizabeth A. Winzeler (42), Philip J. Rosenthal (41), Sergio Wittlin (32), Arjen M. Dondorp (31), François Nosten (30), Kelly Chibale (28), Bruno Pradines (27), and Liwang Cui (22). The top 5 authors in terms of centrality size were David A. Fidock (0.16), Sergio Wittlin (0.14), Didier Menard (0.13), Ric N. Price (0.13), and Elizabeth A. Winzeler (0.10).

**Table 3:** Top 10 publication counts and centralities of authors.

Ranking	Count	Author	Ranking	Centrality	Author
1	74	David A. Fidock	1	0.16	David A. Fidock
2	44	Nicholas J. White	2	0.14	Sergio Wittlin
3	42	Elizabeth A. Winzeler	3	0.13	Didier Menard
4	41	Philip J. Rosenthal	4	0.13	Ric N. Price
5	32	Sergio Wittlin	5	0.1	Elizabeth A. Winzeler
6	31	Arjen M. Dondorp	6	0.1	Philip J. Rosenthal
7	30	François Nosten	7	0.07	Grant Dorsey
8	28	Kelly Chibale	8	0.07	Pascal Ringwald
9	27	Bruno Pradines	9	0.06	Francois Nosten
10	22	Liwang Cui	10	0.06	Liwang Cui

### 4.4 Analysis of research hotspots

#### 4.4.1 The authors’ co-citation analysis

**Figure 5:**
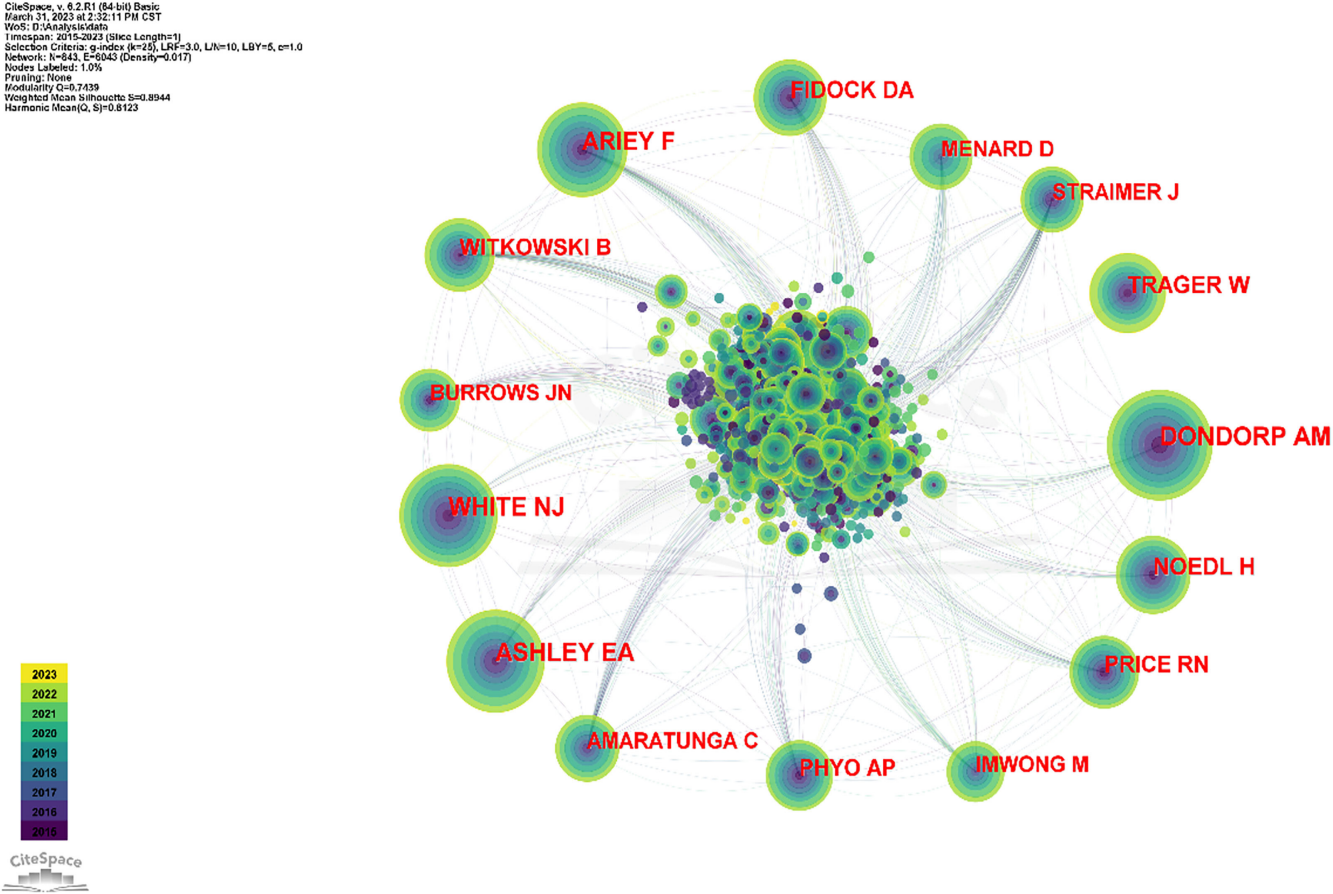
The collaboration network of co-cited authors. The articles published by Dondorp AM, White NJ, and Ashley EA are the most commonly cited.

The software defaults the WHO as author, and this study does not consider the WHO to be among the authors; thus, it was excluded. Some anonymous authors appeared during the analysis, and because such appearance affected the analysis, this part was excluded as well. The top 5 cited authors were Arjen M. Dondorp (634), Elizabeth A. Ashley (564), Nicholas J. White (554), Frédéric Ariey (463), and W. Trager (356). The top 5 co-cited authors for centrality were Timothy J Egan (0.07), Paul M O'Neill (0.06), David A Fidock (0.05), Judith Straimer (0.05), and Christin Sisowath (0.05), (**Table 4** and **Figure 5**).

**Table 4:** Top 10 publication counts and centralities of co-cited authors.

Ranking	Count	Name	Ranking	Centrality	Name
1	634	Arjen M. Dondorp	1	0.07	Timothy J. Egan
2	564	Elizabeth A. Ashley	2	0.06	Paul M. O’Neill
3	554	Nicholas J. White	3	0.05	David A. Fidock
4	463	Frédéric Ariey	4	0.05	Judith Straimer
5	356	W. Trager	5	0.05	Christin Sisowath
6	343	Harald Noedl	6	0.05	Jane Achan
7	307	David A. Fidock	7	0.05	Tomas Paquet
8	286	Benoit Witkowski	8	0.05	Jill M. Combrinck
9	278	Ric N. Price	9	0.04	Markus Rottmann
10	269	Aung Pyae Phyo	10	0.04	Erika L. Flannery

#### 4.4.2 Co-citation analysis of journals

**Figure 6:**
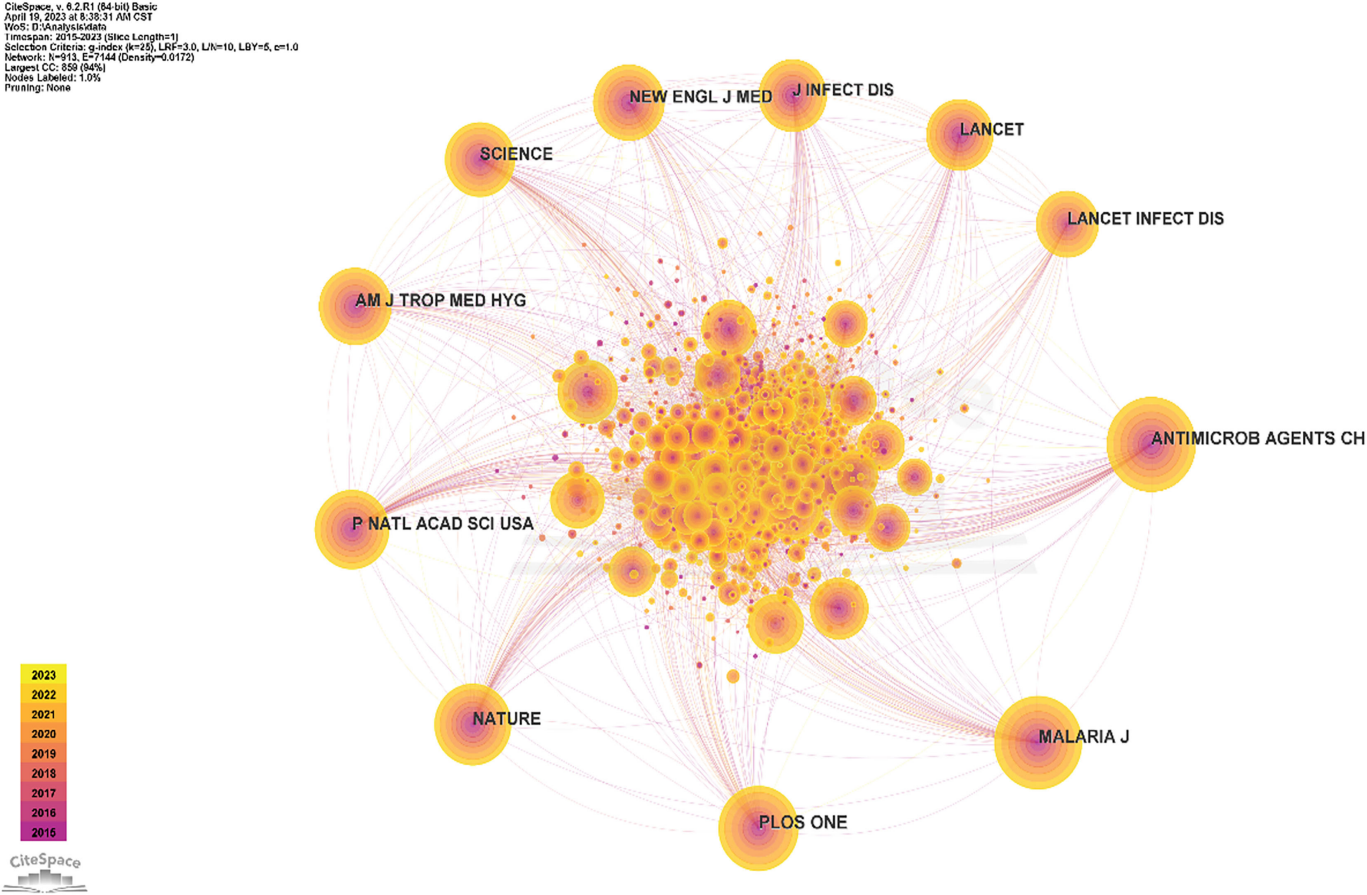
Co-occurrence map of the top 10 co-cited journals.

In the analysis of co-cited journals, the top 5 journals were *Antimicrobial Agents and Chemotherapy* (1,784), *Malaria Journal* (1,784), *PLOS One* (1,495), *Nature* (1,361), and *Proceedings of National Academy of Sciences of the United States of America* (1,284). The top 5 journals for centrality were *Carcinogenesis* (0.36), *International Journal of Nanomedicine* (0.29), *Frontiers in Microbiology* (0.24), *Dalton Transactions* (0.22), and *Journal of Chemical Information and Modeling* (0.21) (**Table 5**, **Figure 6**).

**Table 5:** The top 10 publication counts and centralities of co-cited journals.

Ranking	Count	Name	Ranking	Centrality	Name
1	1,784	*Antimicrob Agents Ch*	1	0.36	*Carcinogenesis*
2	1,784	*Malaria J*	2	0.29	*Int J Nanomed*
3	1,495	*PLOS One*	3	0.24	*Front Microbiol*
4	1,361	*Nature*	4	0.22	*Dalton T*
5	1,284	*Proc Natl Acad Sci USA*	5	0.21	*J Chem Inf Model*
6	1,279	*Am J Trop Med Hyg*	6	0.2	*Drug Develop Res*
7	1,201	*Science*	7	0.2	*J Biomol Struct Dyn*
8	1,192	*New Engl J Med*	8	0.18	*Bioinformatics*
9	1,069	*J Infect Dis*	9	0.17	*AAPS Pharmscitech*
10	1,046	*Lancet*	10	0.17	*Acta Pharm Sin B*

#### 4.4.3 Analysis of co-cited references

**Figure 7:**
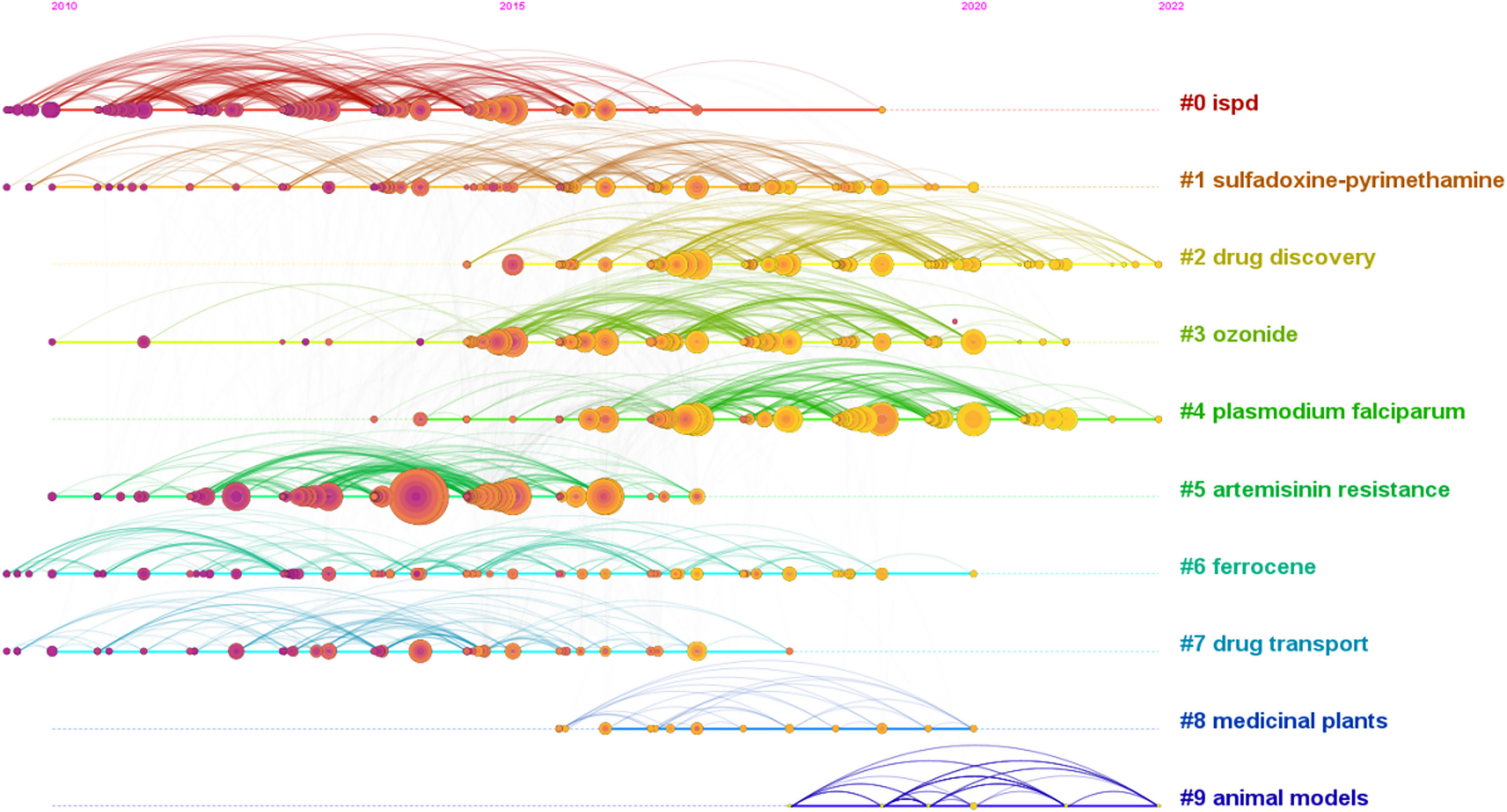
The timeline of co-cited literature showed that the current and future research hotspots in this field may be “new drug development” and “*Plasmodium falciparum*”.

**Figure 8:**
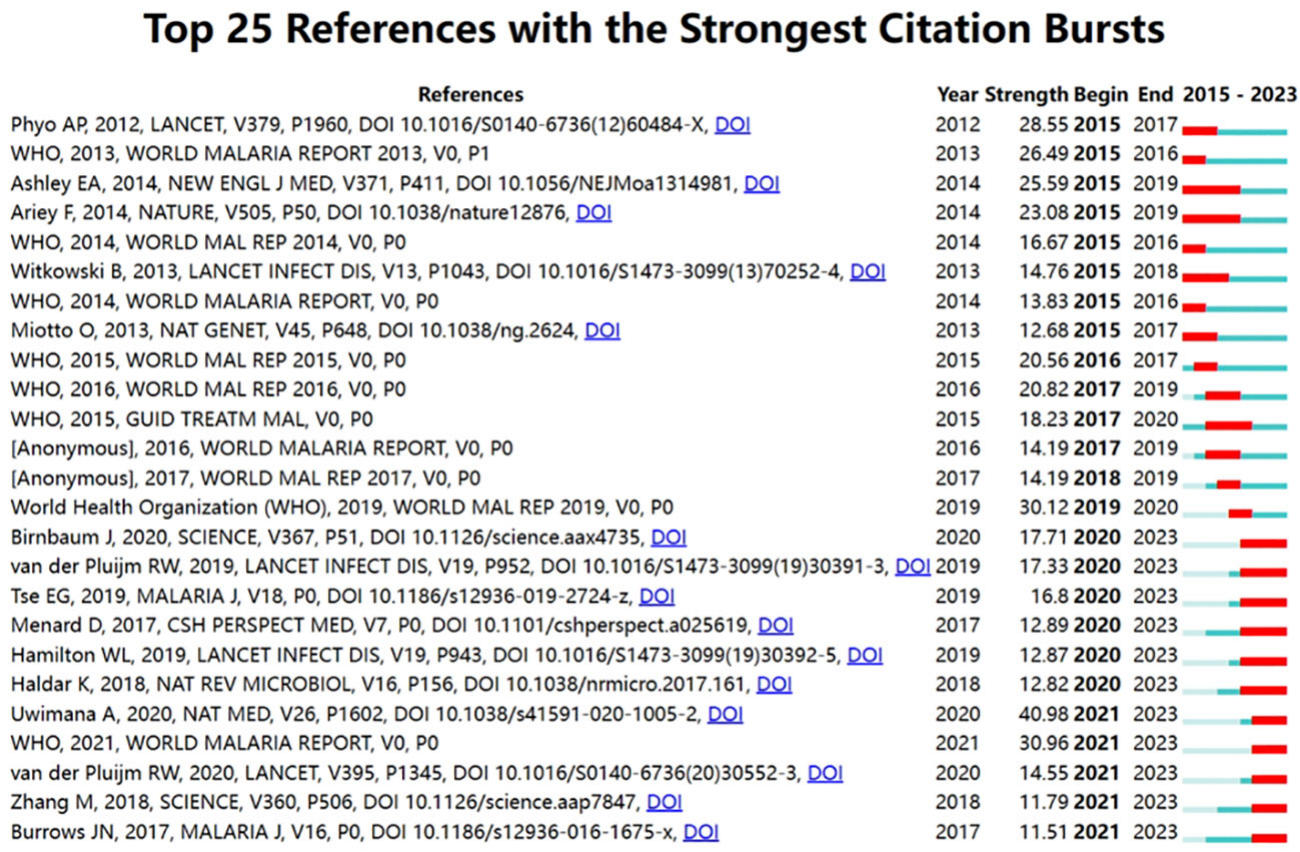
The top 25 most cited references in the field are presented, with the most cited reference being DOI: 10.1016/S0140-6736(12)60484-X.

As shown in **Table 6**, Ashley et al. (2014), Ariey et al. (2014), World Health Organization (2013), Straimer et al. (2015), and World Health Organization (2015) were the top 5 references cited. The *S* values of all clusters were greater than 0.7, indicating that the clustering results are convincing. The first 10 clustering tags (shown in **Table 7**, **Figure 7**) were “ISPD”, “sulfadoxine-pyrimethamine”, “drug discovery”, “ozonide”, “*Plasmodium*”, “artemisinin resistance”, “ferrocene”, “drug transport”, “medicinal plants”, and “animal models”. The timeline graph of co-cited references showed that “drug discovery”, “ozonide”, and “*Plasmodium* “ were the current research hotspots. **Figure 8** shows the top 25 most frequently cited articles.

**Table 6:** The top 10 co-cited reference.

Ranking	Frequency	Author	Title	Year	Journal
1	304	E.A. Ashley	Spread of artemisinin resistance in *Plasmodium falciparum* malaria	2014	*N Engl J Med*
2	275	Frédéric Airy	A molecular marker of artemisinin-resistant *Plasmodium falciparum* malaria	2014	*Nature*
3	170	WHO	World Malaria Report 2013	2013	*Bulletin of the World Health Organization*
4	142	Judith Straimer	K13-propeller mutations confer artemisinin resistance in *Plasmodium falciparum* clinical isolates	2015	*Science*
5	140	WHO	GUID TREATM MAL	2015	*Bulletin of the World Health Organization*
6	128	D. Ménard	A worldwide map of *Plasmodium falciparum* K13-propeller polymorphisms	2016	*N Engl J Med*
7	123	Chanaki Amaratunga	Dihydroartemisinin–piperaquine resistance in *Plasmodium falciparum* malaria in Cambodia: a multisite prospective cohort study	2016	*The Lancet Infectious Diseases*
8	121	Benjamin Blasco	Antimalarial drug resistance: linking *Plasmodium falciparum* parasite biology to the clinic	2017	*Nat Med*
9	117	WHO	World Malaria Report 2019	2019	*Bulletin of the World Health Organization*
10	108	Jeremy N. Burrows	New developments in anti-malarial target candidate and product profiles	2017	*Malar J*

**Table 7:** Top 10 largest clusters of co-cited references.

Cluster ID	Size	Silhouette	Mean year	Top terms (LLR)
0	121	0.866	2013	ISPD
1	111	0.749	2015	sulfadoxine-pyrimethamine
2	106	0.833	2018	drug discovery
3	87	0.805	2017	ozonide
4	80	0.832	2018	*Plasmodium falciparum*
5	79	0.894	2013	artemisinin resistance
6	78	0.894	2014	ferrocene
7	43	0.879	2013	drug transport
8	18	0.988	2017	medicinal plants
9	17	1	2019	animal models

#### 4.4.4 Keyword co-occurrence network analysis

**Figure 9:**
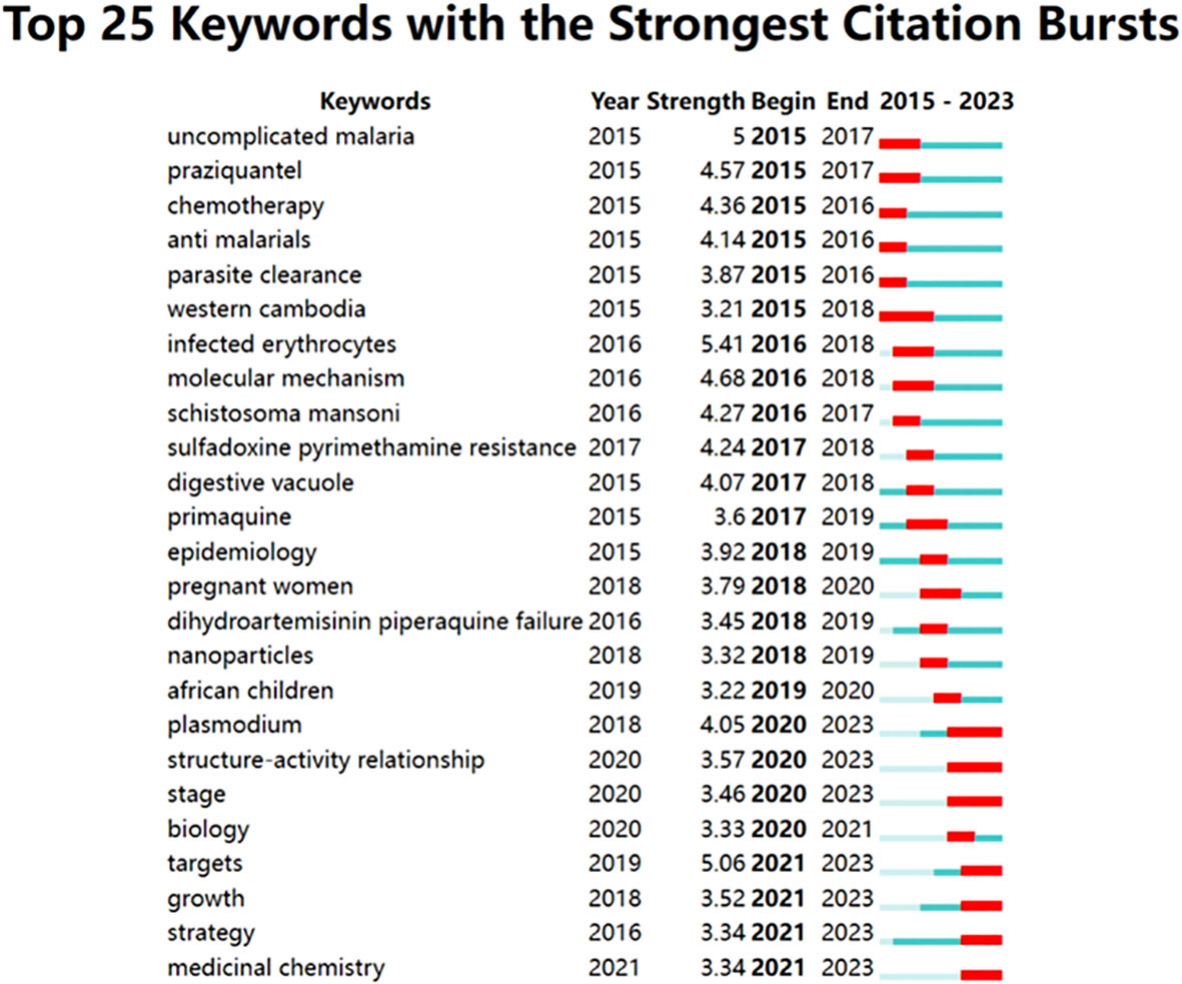
The chart presents the 25 most prominent keywords, their respective duration and intensity of prominence.

The research hotspots in this field were the keywords that appear most frequently in the web co-occurrences. “*Plasmodium falciparum*”, “malaria”, “drug resistance”, “artemisinin resistance”, and “*in vitro* analysis” were high-frequency keywords (**Table 8**, **Figure 9**). The top 5 largest clusters of keywords were antimalarial activity, malaria parasite, artesunate, drug resistance, and artemisia annua (shown in **Table 9**).

**Table 8:** Top 10 keywords by frequency and centrality.

Rank	Count	Keywords	Rank	Centrality	Keywords
1	907	*Plasmodium falciparum*	1	0.22	cation atpase pfatp4
2	557	malaria	2	0.2	mechanisms
3	505	resistance	3	0.19	antitumor activity
4	421	drug resistance	4	0.15	culture
5	420	*in vitro*	5	0.14	vivax malaria
6	388	artemisinin resistance	6	0.11	safety
7	238	antimalarial activity	7	0.1	therapy
8	206	*Plasmodium falciparum* malaria	8	0.1	pfmdr1 gene
9	193	chloroquine	9	0.1	acid
10	188	chloroquine resistance	10	0.1	ferroquine

**Table 9:** Top 5 largest clusters of keywords.

Cluster ID	Size	Silhouette	Mean year	Top terms (LLR)
0	164	0.739	2017	antimalarial activity
1	140	0.611	2018	malaria parasite
2	118	0.694	2016	artesunate
3	115	0.838	2017	drug resistance
4	19	0.948	2016	artemisia annua

## 5 Discussion

### 5.1 Conclusion

Between 2015 and 2023, a variable upward trend was observed in the literature pertaining to antimalarial medication. The USA boasts the highest number of published papers in this area. Alassane Mbengue and colleagues’ research, which has received 384 citations, stands as the most commonly cited paper in this field within the USA. Their publication explicates that PI3P serves as a critical mechanism regarding artemisinin resistance at the cellular and biochemical levels in *P. falciparum* and also identifies PfPI3K as a key target in the fight against malaria (Mbengue et al., 2015). Following this, India, UK, Australia, and other countries are listed.

Oxford University achieved the highest number of publications and the highest centrality ranking, with a total of 184 papers published. These papers delve into the evaluation of resistance activity in various regions, as well as the exploration of resistance mechanisms in *P. falciparum* and *P. vivax* (Pulcini et al., 2015; Yogavel et al., 2018). The study found that Fidock A. David, affiliated with Columbia University Medical Center in the USA, had published the most papers and had the strongest influence among the co-cited authors. He primarily worked on detecting malaria resistance genes in areas susceptible to the disease (Ross et al., 2018), researching drug resistance mechanisms resulting from *P. falciparum* gene mutations (Veiga et al., 2016), and developing novel antimalarial medications through diverse methods (Baragaña et al., 2015).

Moreover, the journal *Antimicrobial Agents and Chemotherapy* was the most co-cited publication. One of its most cited papers provided an overview of the essential characteristics and recent advances in understanding the biology, epidemiology, and clinical significance of malaria (Cowman et al., 2016). Leah S. has discovered a novel tyrosine amide compound, (S)-SW228703 [(S)-SW703]. Imlay et al. (2023) have demonstrated activity in both the asexual blood and hepatic stages (Imlay et al., 2023).

### 5.2 Research hotspot

Through an examination of relevant keywords and significant literature, this paper presents a synthesis of the latest research trends concerning the topic of antimalarial drug resistance:

#### 5.2.1 Antimalarial mechanisms and resistance mechanisms of artemisinin-based combination therapies

Artemisinin-based combination therapies (ACTs) are currently the primary treatment for malaria. The treatment mechanism involves swiftly eliminating both young and mature trophozoites with artemisinin, which is crucial in managing severe conditions and bringing about a cure (Dondorp et al., 2010), while the partner drug kills residual parasites by other means within a few weeks. Furthermore, ACTs exhibit multiple antimalarial mechanisms that contribute significantly towards their effectiveness. Wicht et al. (2020) discovered that the distinctive antimalarial impact of ACTs results mainly from the over-activation of their peroxide bridge by heme, an unavoidable by-product of the parasite’s hemoglobin metabolism in red blood cells. An extensive amount of free radicals could alkylate numerous *Plasmodium* proteins and biomolecules, ultimately causing rapid death of the parasite (Wicht et al., 2020). Artesunate, derived from artemisinin, targets several essential proteins in multiple *P. falciparum* pathways, including REDOX homeostasis, lipid metabolism, and protein synthesis. As such, it exhibits antimalarial effects (Gao, 2023).

Slow clearance of malaria parasites *in vivo* poses a new challenge for ACT treatment. It may jeopardize future global malaria control and elimination efforts. However, resistance to ACT drugs is not entirely comprehensive, the 3-day treatment is variable, and it is unclear whether extending the duration of treatment would boost the clearance rate of the malaria parasite. Presently, there are many ongoing efforts to tackle this predicament, with a primary focus on the resistance mechanism of *P. falciparum* against ACTs drugs. Blood samples were collected from areas where malaria is endemic, and samples were sequenced to obtain the drug-resistant mutant gene of *P. falciparum* using whole genome sequencing (Miotto et al., 2015). Numerous studies have indicated that the *P. falciparum* Kelch13 mutation is a significant factor in the slow parasite clearance rate in ACTs. Aline Uwimana et al. collected cases of pediatric malaria from three locations in Rwanda and observed delayed parasite clearance in over 10% of patients. The prevalence of the Pfkelch13 R561H mutation was significantly greater in patients with recurrent infection than in those who had cleared the infection (Woodrow and White, 2017). Yang et al. discovered that reduced K13 levels hindered hemoglobin breakdown and reduced artemisinin activation (Yang et al., 2019). This aligns with the conclusion by Wicht et al. that heme activation of artemisinin results in a distinct antimalarial mechanism.

#### 5.2.2 Developing new drugs

To overcome the emergence of antimalarial drug resistance, there is an urgent need to develop antimalarial drugs with broad therapeutic potential and new modes of action. In conducting a keyword search, we have discovered that numerous new drugs possess certain antimalarial effects. A study has found that DDD107498 has effective and novel antimalarial activities against multiple life cycle stages of *Plasmodium* parasites, and its molecular target is extension factor 2 (eEF2) (Baragaña et al., 2015); Tanya Paquet et al. found that MMV390048 was effective against multiple stages of the *Plasmodium* life cycle using a whole-cell screen and that its molecular target was the *Plasmodium* parasite kinase phosphatidylinositol 4-kinase (Paquet et al., 2017). The chemical drug DSM265, which has entered the stage of clinical pharmacology research, is a novel dihydroorotate dehydrogenase inhibitor and has the prospect of collaborating with antimalarial drugs for drug development (McCarthy et al., 2017); 2-c-methyl-methyl-erythrin 4-phosphosidal transferase (ispd), an essential and effective antimalarial target, is active against both *P. falciparum* and *P. vivax* (Saggu et al., 2018). MMV008138 was found to selectively target ispd (Ghavami et al., 2018).

Ozonides are one of the most advanced drug classes in antimalarial drug development, aiming to improve the limitations associated with current artemisinin-based first-line therapies, in which peroxide bonds are a key factor in efficacy (Giannangelo et al., 2019). Artefenomel (OZ439), a novel synthetic trioxolane modification, has better pharmacokinetic properties than other antimalarial artemisinin pharmacophores (Phyo et al., 2016), and OZ439 remains effective against most parasite strains expressing mutant K13 in the case of parasite resistance (Straimer et al., 2017). Studies have shown that peroxide antimalarials disproportionately alkylate proteins involved in REDOX homeostasis and that the mechanism of action of these important antimalarials is related to the disruption of REDOX processes. Finding new antimalarial targets is one of the new ways to deal with resistance to existing antimalarial drugs (Siddiqui et al., 2022). These drugs have good pharmacokinetic profiles and acceptable safety profiles, which may become one of the antimalarial drugs of choice in the future.

### 5.3 Research front

Over the past 5 years, research into new antimalarial targets and the development of novel drugs have been the primary focus. As antimalarial drug resistance poses a significant challenge, emphasis is being placed on future development towards tackling this issue. The identification of new drug targets and the subsequent research into discovering effective antimalarial drugs have generated considerable interest within the field. Whole gene scanning and untargeted metabolomics research are currently the popular research methods (Challis et al., 2022). Co-occurrence analysis of the highest-ranked citations shows that “Evolution and expansion of multidrug-resistant malaria in southeast Asia: a genomic epidemiology study” (Hamilton et al., 2019) indicates that drug resistance genes in Southeast Asia have garnered significant attention in this research area.

## 6 Limitation

This paper presents the inaugural bibliometric visualization of antimalarial drug resistance. However, noteworthy limitations exist. The study solely comprises pertinent literature from the Web of Science (WOS) core database, excluding relevant content in other search engines such as PubMed and Google Scholar. As CiteSpace predominantly supports the WOS database, the core database acted as the data source to accommodate the software requirements. Other databases were seldom utilized for the analysis of the software. The software’s limitation to automatically eliminate duplicate entries from distinct databases prohibits its ability to conduct simultaneous analyses on two or more databases. In addition, the study’s merging of near-synonyms cannot completely eliminate possible analysis errors due to subjectivity.

The volume of literature has consistently shown an upward trend every year, signifying a persistent interest within the field. The visual analysis of authors originating from diverse nations, institutions, and journals displays their academic contributions without subjectivity while offering potential collaboration for future research. An overview of literature and keywords within the field reveals research priorities and patterns in antimalarial drug resistance. For instance, developing new drugs represents a current priority, while screening new drug targets using network models could emerge as a future trend. Scholarly peers can reference this analysis to gain insights into the direction of research in this field.

## Author contributions

JZ: Visualization, Data curation, Writing – original draft. MS: Writing – review & editing. MI: Writing – review & editing. HZ: Writing – review & editing, Conceptualization, Visualization.
